# Profiling of Bile Microbiome Identifies District Microbial Population between Choledocholithiasis and Cholangiocarcinoma Patients

**DOI:** 10.31557/APJCP.2021.22.1.233

**Published:** 2021-01

**Authors:** Rungtiwa Dangtakot, Kitti Intuyod, Anucha Ahooja, Jitraporn Wongwiwatchai, Petcharakorn Hanpanich, Aroonlug Lulitanond, Yaovalux Chamgramol, Somchai Pinlaor, Porntip Pinlaor

**Affiliations:** 1 *Biomedical Sciences Program, Graduate School, Khon Kaen University, Nai Mueang, Khon Kaen, Thailand. *; 2 *Cholangiocarcinoma Research Institute, Khon Kaen University, Nai Mueang, Khon Kaen, Thailand. *; 3 *Department of Parasitology, Faculty of Medicine, Khon Kaen University, Nai Mueang, Khon Kaen, Thailand. *; 4 *Department of Radiology, Faculty of Medicine, Khon Kaen University, Nai Mueang, Khon Kaen, Thailand. *; 5 *Center for Research and Development of Medical Diagnostic Laboratories, Faculty of Associated Medical Sciences, Khon Kaen University, Nai Mueang, Khon Kaen, Thailand. *; 6 *Department of Pathology, Faculty of Medicine, Khon Kaen University, Nai Mueang, Khon Kaen, Thailand. *

**Keywords:** Bile microbiome, cholangiocarcinoma, choledocholithiasis, genotoxin, producing E. coli

## Abstract

**Objective:**

Choledocholithiasis (CDL), a potential risk for cholangiocarcinoma (CCA) development, is often a consequence of bacterial infection. Thus, the microbial population that contributes to CDL might also be involved in CCA development. We compared the microbiome in bile fluid of CDL patients and CCA patients.

**Methods:**

Bile samples were collected from CDL (n = 30) and CCA (n =30) patients. Microbial profiling was performed individually by the sequencing of V3-V4 regions of the 16S rRNA gene.

**Results:**

*Enterobacter, Pseudomonas, *and *Stenotrophomonas *species were much more abundant in bile samples from CCA compared to CDL (p<0.05). However, *Cetobacterium*, *Pyramidobacter*, and *Streptococcus* species were less abundant in bile samples of CCA compared to CDL (p<0.05). Although *Escherichia* was predominant in the CCA, *Escherichia coli* itself was more abundant in CDL than in CCA. One CDL case (3.3%) harbored genotoxin-producing *E. coli* as confirmed by PCR. *Enterobacter *and *Pseudomonas *also predominated in CCA according to linear discriminant-analysis effect size.

**Conclusion:**

we demonstrated vast differences between microbial communities in bile of CDL and CCA patients. These bacteria might be partly involved in CCA genesis and may provide novel biomarkers for CCA.

## Introduction

Gut microbiota are involved in host immunity and disease conditions as well as being implicated in the development of hepatobiliary tumors, including cholangiocarcinoma (CCA) (Mima et al., 2017). CCA is a bile-duct cancer that has a significant impact worldwide due to its rising incidence and high mortality rates (Palmer and Patel, 2012; Khan et al., 2019; Sirica et al., 2019). The highest incidence of CCA occurs in the northeastern part of Thailand (Sripa et al., 2007). In Western countries, primary sclerosing cholangitis (PSC) is the most common risk factor for CCA, whereas infection with liver flukes (*Opisthorchis viverrini*) is strongly associated with CCA development in Southeast Asia (Sripa et al., 2007; Palmer and Patel, 2012; Khan et al., 2019).

Previous studies showed that genetic and epigenetic abnormalities of patients with liver fluke-associated CCA are obviously different from those of patients with non-liver fluke-associated CCA (Ong et al., 2012; Chan-On et al., 2013). These suggest that different tumorigenesis processes are occurred in CCA with different etiological factors. Besides PSC and liver fluke infections, other hepatobiliary diseases, particularly choledocholithiasis (CDL), a gallstone in the bile duct which is usually associated with bacterial infection, have also been recognized as risk factors for CCA development (Welzel et al., 2007; Cai et al., 2015). Several studies have been reported that gallstone formation was also observed in patients with *O. viverrini* infection (Riganti et al., 1988; Sripa et al., 2004; Aye Soukhathammavong et al., 2017), highlighting the possible association of microbial dysbiosis in the biliary tract with the pathogenesis of biliary diseases as well as CCA. Changes of the microbial population might alter bacteria-host interactions and eventually to the development of diseases (Blumberg and Powrie, 2012; Cho and Blaser, 2012). Alteration of microbial community has been observed and involved in pathogenesis and progression in variety of diseases including cancer. Although human bile used to be considered sterile, next-generation sequencing has recently shown that the biliary tract harbors a complex microbial population (Pereira et al., 2017; Molinero et al., 2019; Liwinski et al., 2020). Thus, alteration or microbial population in bile might be involved in pathogenesis of hepatobiliary diseases and, perhaps, CCA development. In areas where human liver fluke is not endemic, the biliary tract microbial community of CCA patients was greatly different from that of patients with benign biliary pathology (Aviles-Jimenez et al., 2016; Chen et al., 2019). However, in northeast of Thailand, one of endemic areas of *O. viverrini*, the bile microbial profiles of CCA and CDL have hitherto, not yet reported. In this regard, we hypothesize that bile microbiome of CDL is differed from that of CCA patients and may reflect the possible connection between CDL and CCA development.

Herein, we employed next-generation sequencing to identify bacteria that might be associated with CCA genesis in northeastern Thailand. This study will provide information about the microbiome in the bile fluid of CDL and CCA cases and help to clarify any relationship between bacteria in bile and CCA genesis. 

## Materials and Methods

*Ethics Statement*

Bile samples were the leftover specimens obtained from patients who had enrolled in the research project “Nuclear Magnetic Resonance Spectroscopy of bile from Percutaneous Transhepatic Biliary Drainage”. Informed consent was obtained from all patients. The protocol of this study was approved by the Khon Kaen University Ethics Committee for Human Research based on the Declaration of Helsinki (Ref.# HE591511), and complied with ICH Good Clinical Practice Guidelines. Thirty choledocholithiasis patients (CDL, average age 64.73 ± 14.71; 18 men and 12 women) and 30 cholangiocarcinoma patients (CCA, average age 61.26 ± 10.38; 21 men and 9 women) were enrolled in this study. All patients were diagnosed by ultrasonography and computed tomography. Characteristics of the patients are summarized in Supplement Table 1. 

*Specimen collection*

Bile samples were collected from 30 CDL patients and 30 CCA patients by endoscopic retrograde cholangiopancreatography (ERCP) and percutaneous transhepatic biliary drainage (PTBD), respectively. These procedures were performed by physicians. Then, 500 µl of bile was transferred into sterile 1.5 microcentrifuge tubes and centrifuged at 10,000 rpm for 10 minutes at 4°C. The pellets were kept at -80ºC until used in later steps.

*Preparation of microbial genomic DNA (gDNA)*

DNA was extracted from bile pellets using DNeasy Blood and Tissue Kit (Qiagen, Hilden, Germany) following the manufacturer’s recommendations. DNA concentration was measured using a NanoDrop 2000 spectrophotometer (NanoDrop Technologies, Wilmington, DE, USA). The purity of DNA was checked using the ratio of absorbance values obtained at wavelengths of 260 and 280 nm. Ratios of these values ranging from 1.62 to 2.06 were considered acceptable. DNA integrity was checked by visualizing DNA bands in 1.5 % agarose gel electrophoresis. 

*Detection of the colibactin B (clbB) gene of Escherichia coli*

The presence of *Escherichia coli *and of the* E. coli *clbB gene encoding colibactin B was investigated in all samples using PCR. Primers and conditions of the PCR reactions are shown in Supplement Table 2. A 20 μl PCR reaction consisted of 1x PCR buffer, 1 mM MgCl_2_, 0.3 mM dNTP, 0.25 μM each primer, 0.025 U platinum *Taq* DNA polymerase, and 100 ng of DNA template. *Escherichia coli* strain ATCC25922 DNA were run in parallel as a positive controls. PCR reactions were run in a thermal cycler and an Expand high-fidelity PCR system (BioRad C1000TM Thermal Cycler) and electrophoresed in 1.5% agarose gels, 100 V, 30 min, and visualized under UV light after staining by ethidium bromide.

*Next-generation sequencing*

*Library preparation and sequencing*

In order to check the existence of gDNA of bacteria in bile samples before performing next-generation sequencing, v3-v4 regions of 16S rRNA gene were amplified by PCR from the individual samples. Then, ten samples per group were selected based on quality and quantity as candidates for next-generation sequencing. Sequencing libraries were generated using NEBNext® UltraTM DNA Library Prep Kit (Illumina, USA). Libraries were qualified on the Qubit^@^ 2.0 Fluorometer (Thermo Scientific) and Agilent Bioanalyzer 2100 system. The library was sequenced using an Illumina HiSeq 2500 platform. Finally, 250 bp paired-end reads were generated.

*Assembly of paired-end reads and quality control*

Paired-end reads were assigned to samples based on their unique barcode and truncated by cutting off the barcode and primer sequences. Then, FLASH (V1.2.7) was used to merge paired-end reads based on their overlapping regions. Quality filtering on the raw tags was performed using QIIME (V1.7.0). The tags were compared with the reference database (Gold database) using the UCHIME algorithm to detect chimeric sequences, which were removed.

*Clustering and species annotation of operational taxonomic units (OTUs)*

Sequence analyses were performed by Uparse software (Uparse v7.0.1001). Sequences with ≥97% similarity were assigned to the same OTU. A representative sequence for each OTU was screened for taxonomic annotation using the Mothur software against the SSUrRNA database of SILVA (Wang et al., 2007). 

*Alpha Diversity and Beta Diversity*

Alpha diversity was used to indicate the complexity of the bacterial communities for a sample through indices, including observed-species, Shannon, Chao, Simpson, ACE, and good-coverage. QIIME (Version 1.7.0) was used to calculate these indices. The results were displayed using R software (Version 2.15.3). Beta diversity analysis was used to evaluate differences of samples in species complexity. This was calculated on both weighted and unweighted uniFrac were calculated by QIIME software (Version 1.7.0). 

*Data analysis*

T-tests were used to determine species differing significantly in abundance between groups (p value < 0.05) at various taxon ranks including phylum, class, order, family, genus and species. A T-test was also used to compare the average age of patients between groups. Pearson chi-square was used to investigate differences of gender between the two groups. Linear discriminant analysis effect size (LEfSe) was used to identify bacterial biomarkers for the two groups by LEfSe software. P values <0.05 were considered to be statistically significant. For LEfSE, only taxa with an alpha value of 0.05 and with absolute LDA (log10) scores >3.6 were considered to be statistically significant.

## Results

*The community diversity of bile microbiota in CDL and CCA*

**Figure 1:**
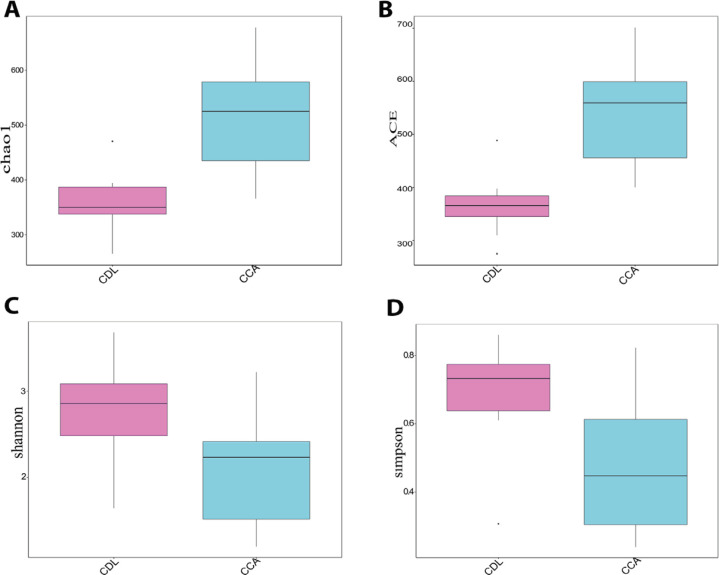
The Richness and Diversity of Biliary Microbiota in CDL and CCA Patients (A-D)

**Figure 2:**
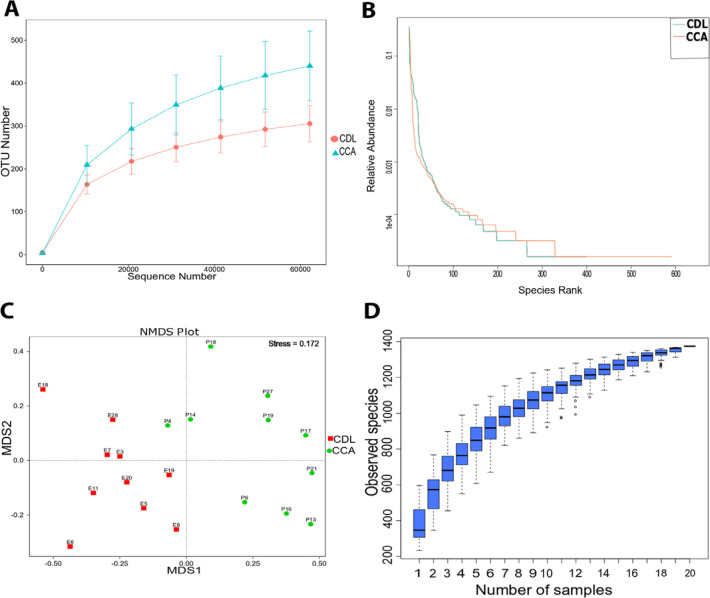
Diversity Curves (A and B) and Differences in OTU Composition between Groups (C). Non-metric multi-dimensional scaling analysis; NMDS (C); Stress value below 0.2: two axes are sufficient to view the data. Species accumulation boxplot (D).

Alpha diversity is an indication of the species diversity of individual samples, measured by the richness of the bacterial community (Chao, ACE) and diversity index (Shannon, Simpson) (Figure 1 A-D). Species richness was higher among CCA patients. However, the diversity index in CCA was significantly lower than among CDL patients (p<0.05), indicating that a few taxa dominated the community in CCA cases. This also indicated by the slope of the rank abundance curve, which is slightly steeper for the CCA samples than for the CDL samples. In contrast, the shallower slope in CDL samples indicated that the abundance of different taxa was similar (Figure 2A-B). The ecological distance of bacteria among groups is displayed by non-metric multi-dimensional scaling analysis (NMDS). The more similar the communities are among samples, the closer their corresponding data points are on the NMDS graph. The NMDS analysis based on OTUs showed that the community of bacteria differed among groups (Figure 2C). A specaccum species-accumulation boxplot showed an initial sharp rise which then levelled out, indicating that adequate sample numbers had been included (Figure 2D).

*Operational taxonomic units of bacteria in bile of CDL and CCA cases*

The 1,978,684 total reads after merging yielded 79,850 tags from the CDL samples and 80,806 tags from the CCA samples. The tags were clustered using a 97% similarity cutoff to obtain an average of 433 OTUs which were classified into 25 phyla, 49 classes, 87 orders, 138 families, and 305 genera of bacteria. The preprocessing statistics and quality control of raw data are shown in Supplement Table 3. The depth of sequencing was 1-75,589 (supplement Table 4). Seven hundred and three OTUs were found in both CDL and CCA cases but differed in number of reads. One hundred and fourteen OTUs were found only in CDL cases. On the other hand, 557 OTUs were observed only in the CCA samples. 

*Distribution and diversity of bacterial taxa in CCA compared to CDL patients*

**Figure 3:**
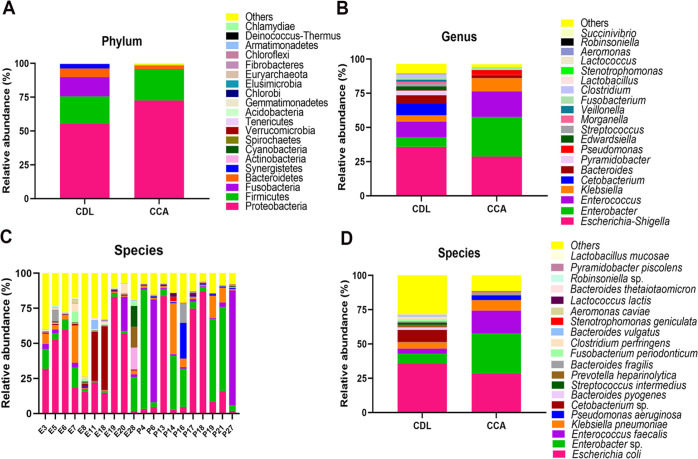
Distribution and diversity of bacteria in bile at level of phylum (A), genus (B) and species (C-D) (top 20). Individual samples for species level are shown in C for CDL (E3-E28) and CCA (P4-P27) cases: aggregated data for species are in D

Distribution and diversity of bacteria for the top 20 taxa (as determined by number of reads) are shown as a bar chart in Figure 3. At the phylum level, diversity was greater in the CDL samples. Proteobacteria was dominant in CCA samples. In the top 20 phyla, Cyanobacteria, Gemmatimonadetes, Acidobacteria, Chlorobi, Fibrobacteres and Armatimonadetes were significantly more abundant, while Fusobacteria and Synergistetes were significantly lower when compared to CDL.

At the genus level, *Escherichia-Shigella* was the most abundant taxon both in CDL and CCA samples. In CDL cases, relative proportions of reads from *Escherichia-Shigella, Enterobacter, Enterococcus, Klebsiella, Cetobacterium, Bacteroides, Pyramidobacter, Edwardsiella* and *Streptococcus* were 35.75%, 7.14%, 11.33%, 4.68%, 8.80%, 5.85%, 3.30%, 2.94%, 1.85%, respectively, whereas *Pseudomonas* was represented by fewer than 0.5% of reads. In CCA cases, *Enterobacter *(28.98% of reads) was the most abundant genus, followed by *Escherichia-Shigella *(28.68%), *Enterococcus *(18.72%),* Klebsiella* (9.72%), *Pseudomonas* (3.57%) and *Bacteroides* (1.99%). *Cetobacterium, Pyramidobacter*, *Edwardsiella* and *Streptococcus* were each represented by fewer than 0.5% of reads (Figure 3B). Among the top 10 genera, *Enterobacter* and *Pseudomonas* were significantly more abundant and *Cetobacterium, Pyramidobacter* and *Streptococcus* were significantly less abundant in CCA cases when compared to CDL.

At the species level, there was considerable heterogeneity among individuals (Figure 3C). *Escherichia coli* (MH755454) was the most abundant species, both in CDL (35.70± 8.21%) and CCA (28.64± 11.71%) samples. On the other hand, *Enterobacter* sp. (MT975285) (CDL: 7.14±7.90, CCA: 28.99±23.24%) and *Enterococcus faecalis* (MT975286) (CDL: 3.82±2.33%, CCA: 16.64±10.18%) were more abundant in CCA, while *Cetobacterium* sp. (MH755457) (CDL: 8.80±16.80%, CCA: 0.21±0.12%) was more abundant in CDL samples (Figure 3C-D). Given the high number of reads from *E. coli* in the 20 samples subjected to next-generation sequencing, we decided to use PCR to confirm the identity of the species in all samples and to determine whether any were producing the virulence factor colibactin (clbB) by PCR. The *uidA* gene of *E. coli* and *clbB *gene were individually amplified from 60 bile samples (30 CDL and 30 CCA) using specific primers. Seventeen CCA samples (56.7%) and 29 CDL samples (96.7%) were positive for the *uidA* gene, indicating the presence of *E. coli* in bile samples of both groups. No CCA bile samples were positive for the *clbB* gene while one CDL case was positive for that gene. 

Statistical analyses of differences in abundance among groups at the species level calculated by dividing the number of reads of a specie from one sample by the total number of reads of that specie from all sample (Supplement Tables 5 and 6). When considering species found in both groups at a relative abundance ≥ 0.5%, two species (*Pseudomonas aeruginosa* (MT890045) and *Stenotrophomonas geniculate* (MT890051)) were significantly more abundant in CCA, while *Bacteroides pyogenes* (MT890048), *Prevotella heparinolytica* (MT890047), *Fusobacterium periodonticum *(MT890049), *Clostridium perfringens* (MT890046) and *Bacteroides vulgatus* (MT890050) were significantly more abundant in CDL cases. However, the group of bacteria was found significant differences only in CDL or CCA had those less than 0.5%.

*Identification of bacteria as biomarker for CCA*

**Figure 4:**
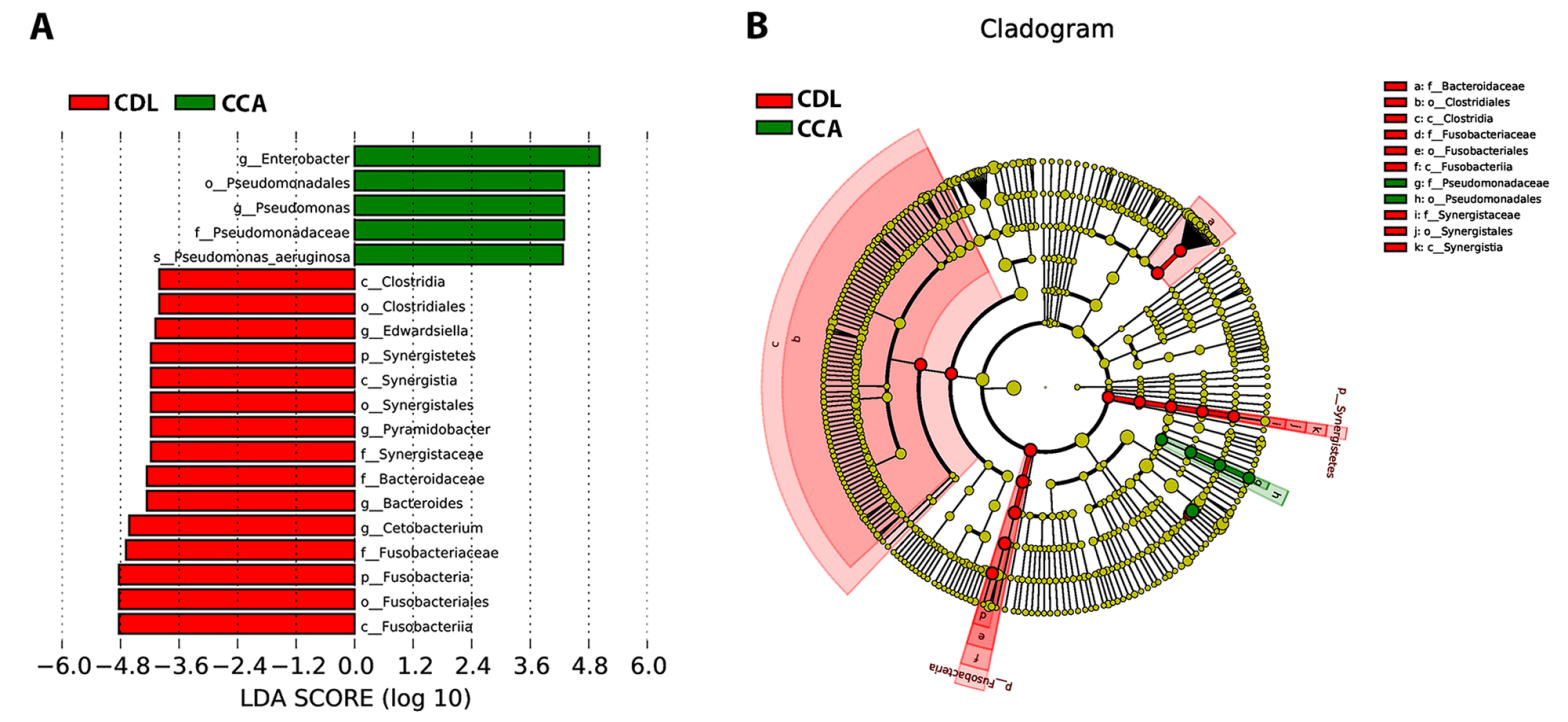
Histogram of the LDA Scores (A) and Cladogram (B) are Shown as Results of LEfSe Analysis for Evaluation of Biomarkers that Differ Statistically Significantly among Groups. c, class; o, order; f, family; g, genus. The letter a-k in the cladogram is the name of bacteria. The levels represent, from the inner to outer rings, genus, family, order, class, and phylum (B)

The linear discriminant analysis effect size (LEfSe) method was used to examine microbial population. The bar graph (Figure 4A) and cladogram (Figure 4B) indicated the taxa between CDL and CCA groups. LEfSe analysis revealed that the relative abundances of 20 taxa could distinguish biliary microbial communities of patients with CDL from those with CCA. In these taxa, 5 and 15 were identified as enriched within patients with CCA and CDL, respectively, confirming these bacterial taxa might be used to discriminate CCA patients from CDL patients.

## Discussion

It is widely accepted that diversity and composition of the microbial community depend on many factors such as ethnicity, geographical provenance, eating habits, etc. (Yatsunenko et al., 2012; Suzuki and Worobey, 2014). In the present study, we compared the microbiomes in bile fluid of CDL and CCA patients who living in endemic area of liver fluke infection using a high-throughput next-generation sequencing (NGS) approach. Several bacterial taxa were commonly found in both groups, but often differed in abundance. Some taxa were found exclusively in either CDL or CCA samples. Bacteria including *Pseudomonas aeruginosa*, *Stenotrophomonas geniculate* and other bacteria that were predominantly or exclusively observed in the CCA group. Interestingly, a strain of *Escherichia coli *which could produce a genotoxin called colibactin was also detected in the bile of one CDL patient but not in any CCA case. In addition, linear discriminant analysis effect size (LEfSe) was used to identify biomarkers for CCA using relative abundance of the different taxa (Segata et al., 2011). Based on this analysis, we suggest that bacteria in the *genera Enterobacter* and *Pseudomonas* may serve as biomarkers for CCA. 

In a liver fluke-endemic area, several reports have suggested that the stone formation might be associated with *O. viverrini* infection (Sripa et al., 2004; Molinero et al., 2019). In this study, microbiome analysis revealed that the abundance of *E. coli* was the highest in CDL patients. Moreover, the abundance of beta-glucuronidase-producing bacteria including* Enterococcu*s sp.,* Klebsiella* sp. *Streptococcus* sp. and* Clostridium* *perfringens* was also higher in CDL cases compared to CCA patients. The high abundance of these bacteria in CDL patients might be involved in stone formation because beta-glucuronidase is an enzyme involving in deconjugation of bilirubin diglucuronide to become unconjugated bilirubin, which in turn combines with calcium, leading to stone formation (Leung et al., 2001). In patients from areas where liver fluke is not endemic, the taxon *Escherichia-Shigella* was the most abundant member of the biliary microbiome both in cases of common bile duct stones and of distal CCA (dCCA). Moreover, abundance of bacteria in the genera *Staphylococcus*, *Okibacterium* and *Corynebacterium* was higher in dCCA patients (Chen et al., 2019). In this study, we also found that *Escherichia-Shigella *was the most abundant bacterial taxon in the bile of CDL patients whereas this position was occupied by *Enterobacter *in the bile of CCA patients. Moreover, *Enterobacter *sp.,* Pseudomonas aeruginosa* and *Stenotrophomonas geniculata* were also more abundant in bile fluids of CCA patients. This is consistent with a tissue-microbiome study of CCA patients in a liver fluke-endemic area (Chng et al., 2016). We also found several bacterial taxa in CDL cases, but not in CCA. Some bacteria can be regarded as opportunistic colonizers. These included *Megasphaera micronuciformis, Actinomyces odontolyticus *and *Prevotella melaninogenica*: all have been associated with infectious disease and the last is a pathobiont promoting chronic inflammation (Kononen and Wade, 2015; Larsen, 2017). 

It is well known that DNA damage is one of the key risk conditions for carcinogenesis. In the case of CCA, this is especially so in association with liver fluke infection (Yongvanit et al., 2012). Bacterial genotoxins, such as colibactin, can cause DNA damage. This genotoxin is encoded by the 54 kb polyketide synthase (pks) genotoxicity island. Pks-containing bacteria mostly belong to the family of Enterobacteriaceae (Nougayrede et al., 2006). Exposure to colibactin has been shown to cause severe genetic damage in mammalian cells and hence, increased rates of gene mutation and tumor growth (Cougnoux et al., 2014). In a mouse model of colorectal cancer, inflammation of the colon promotes the expansion of *Pks+ E. coli*, resulting in colorectal cancer development (Arthur et al., 2012). Not only *E. coli*, other bacteria such as *Klebsiella pneumonia*, *Enterobacter aerogenes* and *Citrobacter koseri* (Fais et al., 2018) have been reported as colibactin-producing bacteria. However, in this study, we only detected colibactin-producing *E. coli* in the bile sample of one CDL case (3.3%) but no CCA cases. Therefore, colibactin-producing *E. coli* might be partly involved in CCA genesis, as similar as previous finding in a mouse model of colorectal cancer (Arthur et al., 2012). Apart from *E. coli*, the abundance of *Enterococcus faecalis* was higher in CDL patients compared to CCA patients, agreeing with previous report in CDL patients from fluke-free areas (Flores et al., 2003). *E. faecalis* might be associated with carcinogenesis partly via stimulating of excessive production of extracellular superoxide (Wang and Huycke, 2007), which could induce double-strand DNA breaks and chromosome instability - a hallmark of cancer (Huycke et al., 2002). In addition to *E. coli* and *E. faecalis*, other bacteria that have been associated with other cancer types, such as* Cetobacterium* sp. (de Carvalho et al., 2019), was also more abundant in the bile of CDL patients. These suggest that the presence of these bacteria in bile of CDL patients might promote CCA development.

In CCA patient, several bacteria were found only in CCA whereas some bacteria were found in both groups but higher in bile samples of CCA patients. Among the identified bacteria at higher abundance in CCA cases, *Pseudomonas aeruginosa* (Markou and Apidianakis, 2014), *Stenotrophomonas geniculate* (Chng et al., 2016) and *Eggerthella lenta* (Woerther et al., 2017) have been reported to be associated with certain cancers. Several bacteria were also observed only in bile samples of CCA. Some species such as *Eubacterium* sp. have been involved in prostate cancer development (Sha et al., 2020). Although several bacteria were identified and postulated to be involved or associated with CCA development, there is hardly any information about the role of these bacteria in terms of causing cancer. Thus, further studies on the functional roles of identified bacterial species and their association with tumorigenesis of CCA are required.

In conclusion, this 16s rRNA-sequencing study of bile found much greater abundance of *Enterococcus *and *Pseudomonas* in CCA patients than in those with choledocholithiasis. *Conversely, Cetobacterium*, *Pyramidobacter* and *Streptococcus* were at lower abundance in CCA cases. These bacteria might be partly involved in CCA genesis and may provide novel biomarkers for CCA. In addition, the presence of colibactin-producing *E. coli *in CDL might increase the risk of CCA development; however, further study is required to confirm its role in CCA genesis.
